# Laccase and Tyrosinase Biosensors Used in the Determination of Hydroxycinnamic Acids

**DOI:** 10.3390/ijms22094811

**Published:** 2021-05-01

**Authors:** Alexandra Virginia Bounegru, Constantin Apetrei

**Affiliations:** Department of Chemistry, Physics and Environment, Faculty of Sciences and Environment, “Dunărea de Jos” University of Galaţi, 47 Domnească Street, 800008 Galaţi, Romania; alexandra.meresescu@ugal.ro

**Keywords:** hydroxycinnamic acids, biosensor, laccase, tyrosinase, enzyme immobilization

## Abstract

In recent years, researchers have focused on developing simple and efficient methods based on electrochemical biosensors to determine hydroxycinnamic acids from various real samples (wine, beer, propolis, tea, and coffee). Enzymatic biosensors represent a promising, low-cost technology for the direct monitoring of these biologically important compounds, which implies a fast response and simple sample processing procedures. The present review aims at highlighting the structural features of this class of compounds and the importance of hydroxycinnamic acids for the human body, as well as presenting a series of enzymatic biosensors commonly used to quantify these phenolic compounds. Enzyme immobilization techniques on support electrodes are very important for their stability and for obtaining adequate results. The following sections of this review will briefly describe some of the laccase (Lac) and tyrosinase (Tyr) biosensors used for determining the main hydroxycinnamic acids of interest in the food or cosmetics industry. Considering relevant studies in the field, the fact has been noticed that there is a greater number of studies on laccase-based biosensors as compared to those based on tyrosinase for the detection of hydroxycinnamic acids. Significant progress has been made in relation to using the synergy of nanomaterials and nanocomposites for more stable and efficient enzyme immobilization. These nanomaterials are mainly carbon- and/or polymer-based nanostructures and metallic nanoparticles which provide a suitable environment for maintaining the biocatalytic activity of the enzyme and for increasing the rate of electron transport.

## 1. Introduction

Phenolic compounds represent a major class of secondary metabolites in plants and are classified into monophenols and polyphenols [1]. Polyphenols are a heterogeneous class of compounds, with important in vitro and in vivo activity [2]. They play a role in food preparation and preservation (quality markers, process and validity date, sensory perception, etc.) and represent an added value factor in the field of marketing [3]. Hydroxycinnamic acids (HC) are structural and functional constituents of plant cell walls and are included in the class of phenolic compounds. Their ability to donate electrons, as well as to stabilize phenoxyl radicals depends mainly on the number and position of hydroxyl groups in their structure [4]. Although the scientific community constantly works to develop easy and rapid methods for the qualitative and quantitative determination of these compounds, the complexity of their chemical structure still causes difficulties in developing detection methods [3,5].

HC determination is considered to be of great importance in characterizing food, as well as nutraceuticals and cosmetics quality. A number of different analytical methods such as gas chromatography (GC) [6], high performance liquid chromatography (HPLC) [7,8], capillary electrophoresis [9], chemiluminescence [10] and the Folin–Ciocalteu spectrophotometry method [11,12] have already been suggested in this respect. Chromatographic methods are accurate, but they require complex extraction procedures, high-purity solvents and qualified staff. Although commonly used, the Folin–Ciocalteu method may lead to an overestimation of the total phenolic compounds due to the fact that the Folin–Ciocalteu reagent reacts with other reducing compounds, which are not phenolic compounds, such as vitamin C [13].

Electrochemical biosensors [14,15,16], characterized by selectivity, portability and rapid response [17,18,19] and requiring minimal sample preparation, most of which are based on the immobilization of laccase or tyrosinase on various support electrodes [20,21,22], are an alternative to HC quantification. The challenge in this case is represented by the correlation of biosensors-based results with those obtained by using classical methods, i.e., the validation of electroanalytical methods [13].

Laccase activity has been applied effectively in nanobiotechnology due to its ability to biocatalyze electron transfer reactions without additional cofactor [23]. Various techniques, such as cross-linking, adsorption, electrodeposition, trapping in a solid matrix [24,25] are used for biomolecule immobilization in order to preserve the enzymatic activity of laccase.

Similarly to laccase, tyrosinase is an oxygenase which catalyzes the oxidation of the o-diphenol group to o-quinone and which is often used for the electrochemical determination of hydroxycinnamic acids [26,27]. Combined biosensors, in which both enzymes, laccase and tyrosinase, are immobilized simultaneously on the same sensitive surface [21,28], have also been used.

The immobilization of an enzyme on electrodes modified with various nanomaterials [29,30] supports further exploration of alternative and advantageous methods for biosensors manufacture [26]. Nanomaterials and nanocomposites are particularly suitable as matrices for enzyme immobilization ensuring good biocatalytic activity, a stable surface for enzyme immobilization and excellent conductivity [31,32,33].

Laccase and tyrosinase biosensors are most often used with high sensitivity and selectivity to mono- and/or diphenolic compounds. They can also be coupled to different devices to perform in situ analyzes, using different detection methods [34], especially voltammetric methods. Laccase and tyrosinase biosensors, depending on their specificity, are used for the detection of phenolic compounds in the food industry [35,36], in the analysis of the environment [37,38,39] or biological samples [40,41]. The activity of these enzymes in the sensitive element of the biosensor, involves the binding and cleavage of molecular oxygen in the active center of enzyme, which include copper ions. This stage is followed by oxidation of the substrate (monophenolase activity) or hydroxylation of monophenols to o-diphenol (diphenolase activity) and oxidation of o-diphenol to quinone [42].

The main challenges in the construction and development of laccase and tyrosinase-based biosensors remain: the choice of nanomaterials used as mediators and the selection of the appropriate method for enzyme immobilization. These issues will be discussed in the sections of this review.

## 2. Hydroxycinnamic Acids, Their Chemical Structure and Their Role in the Human Body

Hydroxycinnamic acids (HC) are phenolic compounds found in fruits, vegetables and beverages (coffee, tea, wine, etc.). Hydroxycinnamic acids are a class of polyphenolic compounds with multiple biological activities. In vitro and in vivo tests have shown that HCs have an important antioxidant, antimicrobial, anti-inflammatory, antidiabetic and even antineoplastic activity [43]. Moreover, HCs are neuro and cardioprotective and also play an important role in the prevention of osteoporosis [42,44,45].

A study conducted in several European countries has estimated the intake of phenolic acids and their food sources [46]. It was found that the main food source of hydroxycinnamic acids was coffee (55.3–80.7% of the total intake), followed by fruits, vegetables, and nuts [24].

Caffeic acid was the main compound present in the daily diet, representing an intake of 188–626 mg/day, and the main food sources were coffee (90.1%), potatoes (2.8%), apples and pears (1.5%). Ferulic acid was consumed in an amount of 44–159 mg/day and its main sources were coffee (54.4%), bread, crispbread and biscuits (27.5%), cereals (2.5%), and fruit juices (1.4%). p-Coumaric acid was present in the diet of subjects in an amount of 11–17 mg/day, through the consumption of coffee (39.2%), wine (11.9%), and various fruits (12%) [46].

Due to the fact that HCs have an optimal bioavailability, an intake of 500/1000 mg/day, through a normal consumption of coffee, bran, citrus, or beer, can have a beneficial impact on human health [43]. In some conditions, the need for HC may be less than the daily intake required. Therefore, the nutraceutical industry offers various phytoproducts or food supplements with high concentrations of HCs, thus enhancing the antioxidant, antidiabetic or lipid-lowering role of these compounds [47].

HCs are often found in olive oils, as well. Studies in the field report the identification of acids such as p-coumaric, caffeic, hydrocafeic, ferulic, synaptic, and cinnamic in olive oils and other olive products [48,49]. These compounds are found in different proportions in olives, according to their variety, degree of maturity, climate, harvest time, and even to the technological process to which olives are subjected. HCs are related to the benefits of olive oil, having an important role in the quality of olives by influencing the organoleptic elements and identifying the optimal harvesting and consumption (maturation) time. Moreover, their antioxidant activity contributes to olive oils preservation and HCs quantification may represent a qualitative parameter due to its correlation with the peroxide index, the amount of free fatty acids and sensory quality [48,49].

Especially due to their antioxidant action, HCs have also been used in the cosmetics industry. For instance, ferulic acid is often found in antiaging products, being an active ingredient that supports intracellular antioxidant defense systems [50] but also in depigmenting cosmetics, due to its ability to inhibit the main enzyme of melanogenesis [51].

Ferulic acid is found in concentrations between 0.5 and 1% in various cosmetic formulations such as face masks, creams, or antioxidant or moisturizing lotions. In beauty salons or medical offices, a higher concentration of ferulic acid (12%) can be used in combination with vitamin C and hyaluronic acid [51]. Some research shows that the use of ferulic acid in certain concentrations can alleviate atopic dermatitis [52,53].

These benefits brought by ferulic acid on the appearance of the skin, have influenced the cosmetics industry, in order to permanently improve the products.

**Figure 1:**
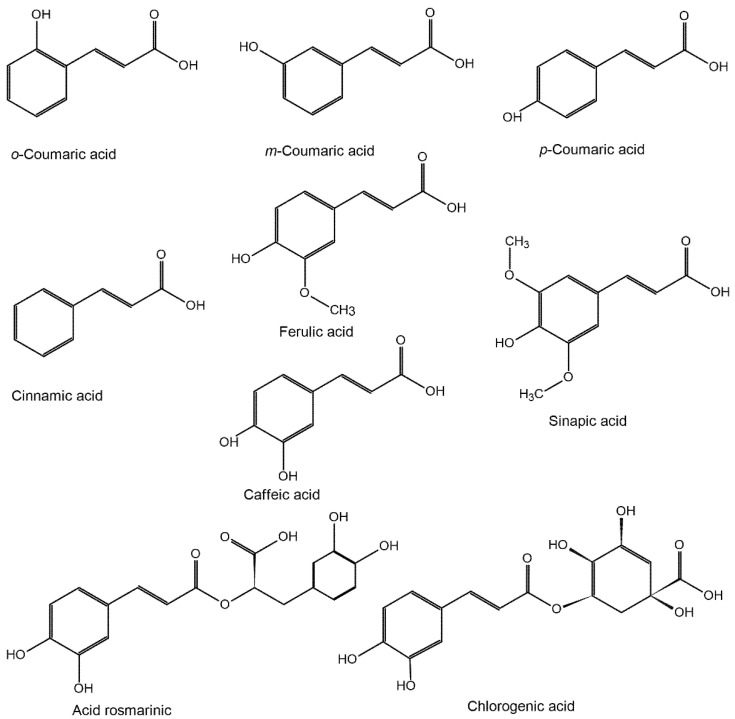
The structure of hydroxycinnamic acids and of their related compounds.

As regards plants, HCs may be found either freely (monomers or dimers) or, more frequently, conjugated (esters of carboxylic acids or sterols, amides, or amines, glycosides of mono- or disaccharides) and insolubly bounded (attached to the structural components of the plant cell wall) [54]. The chemical structures of the compounds belonging to the HC family are shown in Figure 1.

The HCs family includes coumaric acids (CoA), caffeic acid (CA), ferulic acid (FA), cinnamic acid (CnA), synaptic acid (SA), and their derived compounds. CA is present in plants, mainly in the form of 3-, 4-, or 5-cofeil esters of quinic acid, which is called chlorogenic acid (ChA). Rosmarinic acid (RA) is an ester of CA and 3,4-dihydroxyphenylacetic acid. Dimeric derivatives of HC may form in plants by coupling their monomeric precursors. During food processing, highly volatile 4-vinyl derivatives of HC form by thermal decarboxylation during high temperature treatments (e.g., roasting coffee beans) or by enzymatic decarboxylation during fermentation. A significant increase in plasma antioxidant activity (AA) was observed in humans after coffee consumption [55,56], which is one of the main dietary sources of HC (ChA is the most abundant). It is believed that the physiological functions of HC are mainly caused by AA and by the AA of their metabolites.

For example, the ability of FA ethyl ester to suppress the induction of lipid peroxidation and protein modification, thus preventing oxidative stress and toxicity, has been confirmed in cell cultures [57]. HCs may support the elimination of oxidative damage acting directly on reactive species or protecting the endogenous antioxidant system. In this respect, phenolic compounds have various effects, such as radical elimination, metal chelation, and modulation of enzymatic activity, as well as altered signal transduction pathways [58,59]. Structural characteristics, such as aromatic substitution, glycosylation, and conjugation with other phenols or organic acids are considered the main factors determining their ability to act as antioxidants [54]. Nevertheless, physico-chemical parameters (dissociation energy of the O–H bond, electrochemical properties, acid–base properties, partition properties, and nature of reactive species) and environmental characteristics (medium/solvent, pH, presence of other antioxidants or pro-oxidants) are also important [54].

**Figure 2:**
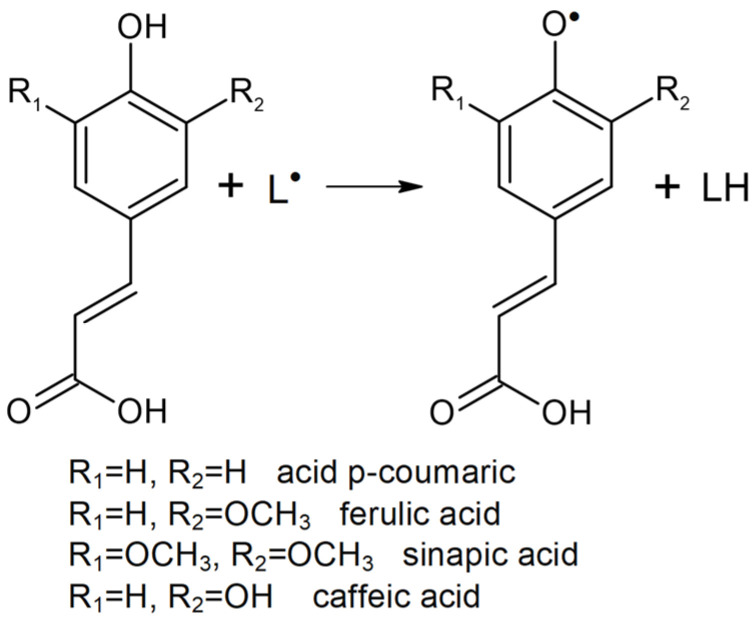
The mechanism of free radical scavenge by HCs.

The specific chemical structure of HCs (a phenolic ring and an extended side chain) allows them to successfully eliminate free radicals (L^•^), thus acting as radical chain disrupting antioxidants (Figure 2). Generally, there are two reaction mechanisms in radical scavenging [60]. The phenolic group (-OH) in an HC may extract a hydrogen atom (hydrogen atom transfer reaction mechanism) or an electron (electron transfer reaction mechanism), thus forming a free radical phenoxyl stabilized by electrons delocalization (AO^•^).

**Figure 3:**
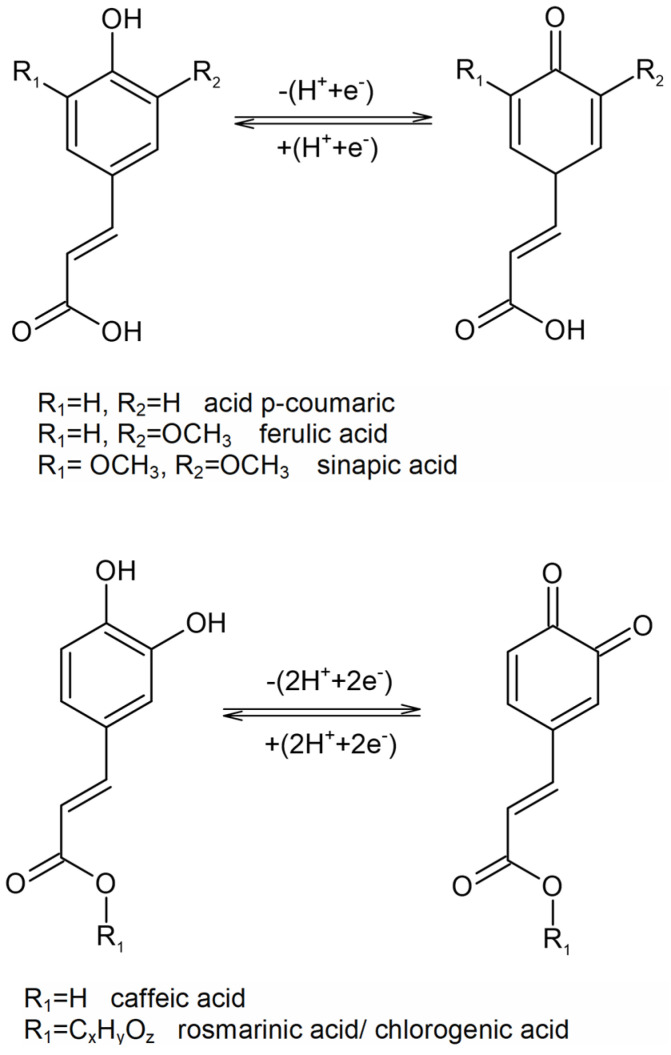
The redox processes of HCs at the sensitive element of the biosensors based on tyrosinase or laccase.

The latter mechanism could be described either as sequential electron transfer with proton losses, or as electron transfer followed by proton transfer [54]. As far as ortho-diphenols are concerned, the fact has been demonstrated that the transfer of two electrons per molecule conditions the subsequent formation of an ortho-hydroxyphenoxyl radical (the first electron transfer) and of orthoquinone (second electron transfer) (Figure 3) [61,62].

The AA correlation of HCs with their redox potential (E_pa_) has shown that this parameter could be considered a good criterion for measuring their antioxidant activity [61]. The higher the number of hydroxyl groups on the aromatic ring, the lower the E_pa_, while the methoxyl group instead of the hydroxyl group on the phenyl ring shifts the potential to positive values. The side chain with a double bond conjugated to the aromatic ring also affects the E_pa_ of HC [54]. Tyrosinase and laccase catalyzes oxidation of various phenolic compounds. Tyrosinase oxidizes mainly p-monophenols and o-diphenols to quinones, whereas laccase oxidizes a larger variety of compounds, such as substituted mono- and polyphenols, aromatic amines and thiol. The reaction products resulted by means of interaction between substrates and tyrosinase could be electrochemically detected by voltammperometric biosensors [63].

## 3. General Notions Regarding Enzymatic Biosensors—Types and Classifications

Electrochemical biosensors are analytical devices similar to electrochemical sensors, which incorporate biological molecules for rapid and accurate detection of target species [64,65]. More precisely, biosensors are chemical sensors which use the recognition properties of biomolecules on the electrode surface. Such sensors have been widely used to determine biological molecules, pathogens or tumor markers, highly relevant in the medical field [66].

Taking into account the ways in which enzyme-based amperometric biosensors may be used Castillo et al. classified them as follows [67]:

- “off-line” devices—analytical equipment based on biosensors which detect target analytes in previously collected biological samples. For example, commercial devices for measuring blood glucose [68].
- “in vivo” devices—implanted biosensors which detect, in real time, the extracellular changes regarding the concentrations of the envisaged analyte. Given the invasiveness of these implantable devices, their use is limited, mainly to preclinical animal research [69].
- “on-line” devices—integrated biosensors which have a sampling device implanted in the body or in the biological material. For example, microdialysis probes may be implanted and connected to a flow through a detector which incorporates a biosensor [70].

Carbon-based nanomaterials are commonly used in electroanalysis thanks to their beneficial properties (good biocompatibility, chemical stability, signal reproducibility, rapid electron transfer, and high thermal and electrical conductivity) and to their possibility to combine with/be modified by various other nanomaterials or mediators at rather low costs [71,72].

**Scheme 1:**
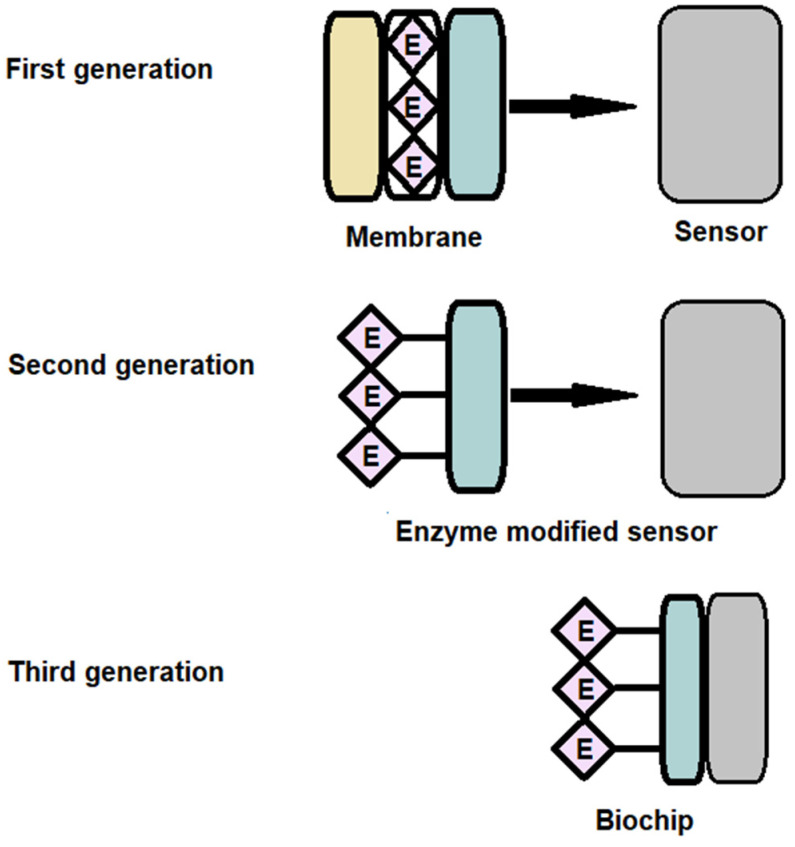
The classification of the biosensors according generation criteria.

Amperometric enzymatic biosensors are divided into three main classes or generations, depending on the method of electron transfer used to quantify the biochemical reaction [73] or the biosensor components degree of separation (transducer, enzyme, mediators and cofactors). In the case of first generation biosensors, the biocatalyst is attached to the membrane surface, then fixed to the transducer surface. In the second generation of biosensors, the semipermeable membrane is removed, and the enzyme is adsorbed or immobilized directly on the surface of the transducer. The third generation refers to the miniaturized biosensors when the biocatalyst is directly connected to the electronic device that transduce and amplifies the biosensor response. The classification of biosensors according to this criterion is presented in the Scheme 1.

Since the presence of the enzyme is indispensable in all cases, sensor performance is conditioned by various parameters, such as pH and working temperature. First-generation biosensors measure the concentration of analytes and/or of enzymatic reaction products, which diffuse to the surface of the transducer and generate an electrical response. They are also called amperometric biosensors without a mediator [70].

In the case of such biosensors, the enzyme is immobilized on the surface of the transducer, and its ability to transform a substrate into an electroactive, measurable compound is exploited [74]. These biosensors are based on enzymes which belong to two main categories: oxidases and dehydrogenases both of which requiring the presence of coenzymes during catalysis (e.g., nicotinamide adenine dinucleotide, nicotinamide adenine dinucleotide phosphate, adenosine triphosphate, and flavin adenine dinucleotide), which must be reactivated in order to allow the enzyme to catalyze further reactions [75,76].

First-generation biosensors have been proved to be highly sensitive and characterized by a very short response time, usually about one second [77]. However, such biosensors often require an electrode pretreatment stage in order to generate a reproducible surface and an optimal response [78]. In addition, the repeated use of amperometric biosensors, especially in complex biological matrices or undiluted samples, often leads to the deterioration of the sensitive surface [79], affecting the biosensor response.

Second generation biosensors use mediators, as oxidizing agents, with the role of electron carriers [80]. This approach makes it possible for the biosensor to operate at a low potential, avoiding O_2_ dependence and the impact of interfering molecules. The most common and best-known mediators are potassium ferricyanide, ferrocene, methylene blue, phenazines, Prussian blue, thionine, toluidine blue, and inorganic redox ions [81]. Mediators may be added to the sample or immobilized on the electrode surface. In order to immobilize the mediator, it must be captured as close as possible to the enzyme [82]. The characterization of the biosensor may be done by measuring the time required to consume the mediator. Suitable mediators are stable during the reaction and recover after the electron transfer. Moreover, the mediator should have a lower redox potential than other electroactive compounds in the working sample [81]. Second-generation biosensors are less used than first-generation ones because they have lower stability due to the immobilized mediator.

A third-generation biosensor consists of three elements: the enzyme, as a bio-recognition element, a polymer with redox properties which ensures the propagation of the signal and the electrode as a support surface [79]. The polymer with redox properties has the role of “connecting” the redox center of the enzyme to the surface of the electrode, aspect which improves performance. Third-generation biosensors are a promising, but still evolving option [70].

Unlike other biosensors based on antibodies, nucleic acids or biological cells or tissues, enzymatic biosensors uses the enzyme as a recognition element. It is immobilized on/into the support on the transducer surface by various methods, so that the enzymatic activity is maintained and maintained for as long as possible. This biosensors based on enzymes have been studied and developed a lot in recent decades, precisely because of the advantages it offers, such as: high sensitivity and specificity due to favorable enzyme-substrate interactions, the high turnover rates of biocatalysts (for example the product of catalyst activity and lifetime), portability and miniaturization possibilities for rapid testing and diagnosis, which make them of great interest for clinical analysis, disease monitoring or food control.

Furthermore, the principle of detection of the enzymatic biosensor allows the detection of analytes by measuring several types of changes that may occur (redox reactions, gas release or absorption, proton concentration, and heat emission) during substrate consumption or product formation following a enzymatic reactions. This makes enzymatic biosensors suitable for determining a wide range of analytes in areas of activity of great importance [83].

## 4. Enzyme Immobilization Strategies for the Development of Enzyme Sensors

The choice of an appropriate immobilization technique is essential for the manufacture of biosensors [84]. One of the simplest methods of immobilizing the enzyme is by physical adsorption. Practically, the enzymatic solution comes into contact with the electrode surface for a specific period of time and afterwards the unabsorbed biomolecules are removed by washing [85]. Nevertheless, this method may have disadvantages due to the weak or reversible binding between the enzyme and the support [86].

Enzymes may also be immobilized by incorporation into three-dimensional matrices, such as an electropolymerized film [87,88], a silica gel [89], or a carbon paste [90]. This strategy is simple and the enzyme activity is not affected by the chemical reaction with the monomer, being possible for the enzyme to be released through the porous matrix. Such a strategy has been applied for the development of a laccase—based biosensor for the detection of phenolic compounds. Laccase was immobilized during the electrodeposition of a thin film of polydopamine on the surface of a carbon electrode. Following morphological analyzes, it was shown that the enzyme was uniformly immobilized, and the redox behavior of the polymer and the activity of the laccase were not negatively influenced [37].

Crosslinking is another very commonly used immobilization technique, but it involves the use of a reagent, such as glutaraldehyde [53,91]. The disadvantage is that crosslinking may decrease enzyme activity due to the changes in the three-dimensional structure of the heteroprotein.

In a recent study, this technique was used to immobilize tyrosinase using glutaraldehyde on a screen-printed carbon electrode modified with gold nanoparticles. Morphological analysis have shown the groups of proteins firmly anchored to the network of gold nanoparticles, thus ensuring a favorable electrical connection with the enzyme [26].

In another paper studying the detection of caffeic acid, a polyvinyl alcohol (N-methyl-4 (4′-formylstyryl) pyridinium acetosulfate methyl) was used as a crosslinking agent for the purpose of immobilizing laccase, not before immobilizing gold nanoparticles on screen-printed electrodes based on graphene and multilayer carbon nanotubes, using the same photopolymer [92].

Similar to the previous study, gold nanoparticles were used as the basic modification of the support electrode. Given the precise results obtained, we can consider that the gold nanoparticles improve the electron transfer between the enzyme and the support electrode, avoiding the decrease of the enzyme activity. Covalent enzyme binding of is another conventional immobilization method which is accomplished by an initial activation of the support with the help of a coupling agent, and a subsequent binding of the enzyme to the activated surface. Coupling with glutaraldehyde [93] and carbodiimide [94] are among the most exploited techniques for covalent attachment of enzymes to different matrices [86].

Enzymes may also be immobilized by affinity bonds between a functional group of the support (e.g., avidin, lectin, and metal chelates) and a specific group (e.g., biotin, carbohydrates, and histidine) [95] naturally present or genetically modified, at a specific location in the enzyme sequence which does not affect biocatalytic activity [96]. Affinity interactions allow the creation of highly ordered structures with high biosensitivity [86].

Several combined immobilization strategies may be used to develop enzymatic biosensors. For example, the enzyme may be pre-immobilized by adsorption, covalent bonding or affinity bonds before being incorporated into a porous polymer [97] or a carbon paste [98].

The immobilization process should be reproducible, simple, with a low cost and the shortest processing time. The choice of the most appropriate enzyme immobilization technique for the development of a sensitive and stable biosensor depends on the nature of the enzyme, the transducer and the mode of detection [99]. Irrespective the method of immobilization, enzymes must retain their biological activity and not be desorbed during the operational use of the biosensor. The sensitivity (limit of detection) and selectivity of biosensors are influenced by the accessibility and activity of the immobilized enzymes. The covalent bond allows for a better stability as compared to the other immobilization methods. Improved accessibility may be achieved with the help of an electroactive mediator. Randomly targeted enzymes may lose their ability to bind to the substrate. Enzymatic immobilization may be better controlled by prior chemical modification of enzymes with markers such as biotin, histidine, or thiol groups [100]. Optimized immobilization includes the correct and uniform orientation of the enzyme with minimal modification of the enzyme structure. A more obvious enzymatic activity is obtained by means of a uniform and correctly oriented immobilization as compared to the adsorption method which can lead to the decrease of the enzymatic activity [86].

The performance of a biosensor is obviously directly influenced not only by the enzyme and the immobilization method, but also by the transducer and the detection method. Moreover, from a practical point of view, the interference of the components in the sample to be analyzed is an important factor which influences the biosensor performance [101].

Although enzymes are generally very specific and selective, electrochemical detection is influenced by electroactive species (e.g., caffeic acid, chlorogenic acid, ascorbic acid, etc.) present in biological samples.

**Figure 4:**
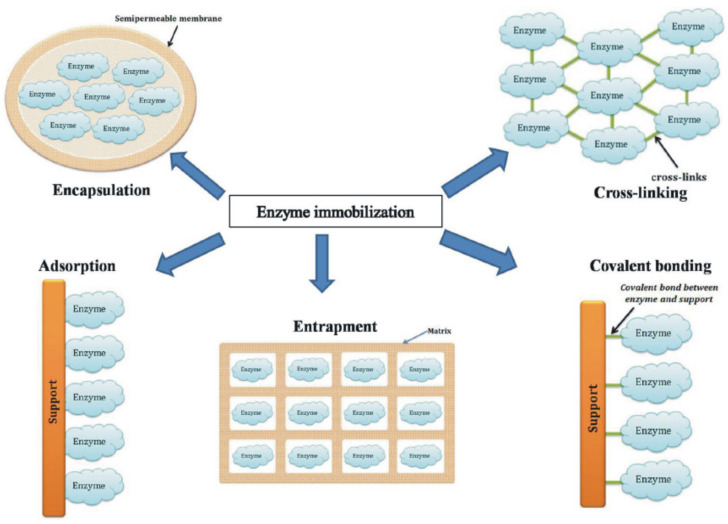
The main techniques of enzyme immobilization. Reprinted from [100] with permission of publisher.

Despite the numerous immobilization methods available, it is important that a suitable technique should be chosen, which may be adapted not only to the enzyme used, but also to the particularities of the biomolecule to be detected [86]. Figure 4 shows the most used enzyme immobilization techniques.

Enzymatic biosensors could have some limitations related to loss of enzymatic activity upon immobilization of enzyme and use of biosensor, as well as difficulties in recovering biosensor activity. These drawbacks can be avoided by using optimal enzyme immobilization techniques [102]. However, enzyme immobilization, in many cases, involves decreased catalytic activity because there are additional stages of pretreatment of enzyme solutions [99].

For example, the immobilization techniques can have some disadvantages. Enzymes immobilized by this adsorption method can be more easily modified by changes in experimental conditions, such as temperature, pH or ionic strength, which alter the tertiary structure of the protein of the enzyme [103].

Moreover, nonspecific adsorption of other compounds on the electrode surface can contaminate the sensitive material, leading to interference in the biosensor response. Immobilization of the enzyme by polymerization is another technique often used, which may also have limitations. Although polymerization uptake gives enzymes high stability and minimizes their loss, the gel matrix used can interfere with the deep diffusion of substrates to the active site of the enzyme, decreasing its activity. That being said, we believe that immobilization methods, although diverse and relatively simple to perform, can cause enzyme changes and decreased analytical performance of biosensors. Therefore, in order to choose the optimal technique, the type of enzyme and nanomaterials used in the construction of the device must be taken into account. Ongoing studies are needed to improve experimental results and reduce the difficulties that may arise [83].

## 5. Enzymatic Biosensors for the Detection of Hydroxycinnamic Acids

Hydroxycinnamic acids are often determined by using the enzymes tyrosinase and laccase which modify the electrodes due to their ability to oxidize these compounds [104]. The oxidation mechanisms are different, their result being represented either by quinones, from simple oxidation, or by compounds which contain several oxygen atoms.

The catalytic role of enzymes allows the amplification of electrochemical signals obtained by reducing the oxidation products of phenolic analytes. Therefore, they may be considered components of the electrochemical sensor. The process of substrates recognition is influenced by enzyme activity. Enzymes are also recognition elements for the molecules of interest, and the selectivity of their substrate leads to the detection of specific molecules [105], such as HC.

Enzyme immobilization on the surface of the electrodes is a technique intensively studied and practiced, but there may still be some difficulties which require innovative solutions. For a correct immobilization of an enzyme several aspects must be taken into consideration: compatibility with the advanced materials used in the development of the biosensor, changes in the conductive properties of the electrode surface, avoidance of enzyme inactivation at the catalytic site [106].

### 5.1. Tyrosinase-Based Enzymatic Biosensors

Tyrosinase (Tyr) is a metalloenzyme, which possesses, at the level of the enzymatically active site, two copper ions, each of them being coordinated by a number of three histidine residues in the enzymatic polypeptide chain [107]. Reversible electron transfer via copper ions (Cu^+^; Cu^2+^) gives the enzyme an oxidoreductase action. The two copper atoms of the enzyme bind to atomic oxygen, thus catalyzing the two reactions of melanin synthesis: the hydroxylation reaction of a monophenol and the conversion of an o-diphenol to an o-quinone [108,109].

According to chemical and spectroscopic tyrosinase studies, its active site contains a coupled binuclear copper complex. Tyr has a type 3 copper center. The oxygenated form has two tetragonal Cu atoms (II), each being coordinated by two strong axial N-His ligands and a weaker, axial one. The exogenous oxygen molecule binds as peroxide and binds the two Cu centers. The electronic structures are explained by the complexes of the cupric model which are terminal (*cis* geometry-1,2) or lateral.

**Figure 5:**
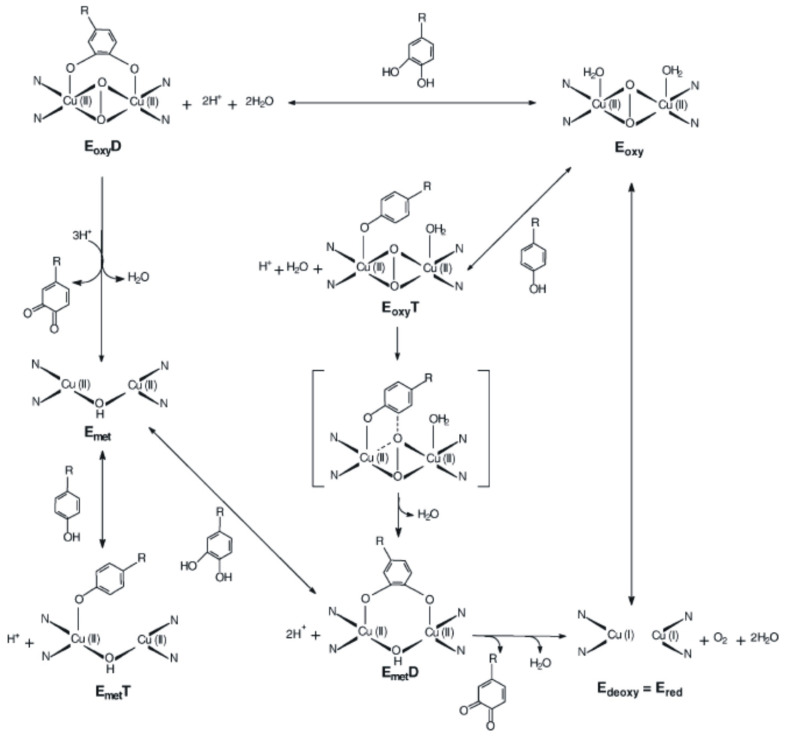
The catalytic cycle for the oxidation of monophenols and diphenols to o-quinone derivatives by tyrosine in the presence of oxygen Reprinted from [110,111] with permission of publisher.

Met-tyrosinase (E_met_) contains two tetragonal Cu (II) ions antiferromagnetically coupled through an endogenous bridge. Deoxy-tyrosinase (E_deoxy_) has a bicupric structure (Cu (I)–Cu (I)). These three copper states in the active site of tyrosinase suggest a structural model for the reaction mechanism involved in o-hydroxylation of monophenols and in the oxidation of the resulting diphenols, as shown in Figure 5 [110,111].

Tyrosinase is a natural enzyme which may be obtained from multiple sources such as bacteria, fungi, plants and mammals and may be purified relatively easily. Various microbial strains such as *Streptomyces glaucescens, Agaricus bisporus,* and *Neurospora crassa* have been used to produce tyrosinase.

Due to its ability to react with phenols, tyrosinase is useful for various applications in food, biomedical and pharmaceutical industries. Tyrosinase may be useful for water or soil decontamination in the biosynthesis of L-DOPA (L-3,4-dihydroxyphenylalanine), a drug highly recommended for patients with Parkinson’s disease [112], but may also be used as a food additive due to its crosslinking properties during food processing [113]. The melanin pigment formed under the action of tyrosinases provides good protection for mammals and against UV radiation [114].

Ty-based electrochemical biosensors may be considered “classics” because Tyr is an enzyme suitable for evaluating the electrochemical properties of materials, with the help of which new nanostructured electrodes are developed [35,115,116]. Nanomaterials designed to improve the performance of tyrosinase-based biosensors include carbon nanostructures (e.g., carbon nanotubes, graphene, carbon quantum dots, and nanodiamonds), metal nanoparticles, and oxides (e.g., gold, platinum, and nickel oxide nanoparticles) and semiconductor nanoparticles (e.g., CdS quantum dots and titanium nanoparticles) [16,39,117,118,119].

Their large surface area, the presence of numerous pores and their thermal stability are characteristics which increase the performance of biosensors, especially with the transfer of electrons between the immobilized enzyme and the electrode surface. Gold nanoparticles (AuNPs), for example, are able to transfer electrons between the active centers of enzymes and the electrode, as well as to perform direct electron transfer (DET). AuNPs also have such advantages as biocompatibility and low cytotoxicity [120,121].

Such an AuNPs-modified screen-printed electrode (AuNPs-SPCE) was used as a support for tyrosinase immobilization (Ty-AuNPs-SPCE) by crosslinking with glutaraldehyde for the analysis of caffeic acid in 15 commercial beers, by direct amperometry. A solution of 50 U·μL^−1^ tyrosinase was prepared for the enzymatic modification of the sensor by dissolving the lyophilized powder in an appropriate volume of 0.1 M phosphate buffer with pH 7.4. AuNPs-SPCE was modified by sequentially pouring 5 μL of enzyme solution, allowing the surface to dry gradually, then 5 μL of 25% aqueous solution of glutaraldehyde was added to the surface of the working electrode for crosslinking. After about 30 min, the obtained biosensors were washed with water and stored for further analysis.

All the important parameters were optimized for fast and reliable detection of phenolic compounds in complex beer samples. Caffeic acid was found among the analyzed compounds, obtaining good analytical and kinetic results (LOD = 2.3 μM), according to domain-specific studies. The final goal was to quantify the total phenolic content of the tested beers and to validate the amperometric method with the classical Folin–Ciocalteu spectrophotometric method [26].

Screen-printed electrodes based on carbon nanofiber modified with gold nanoparticles (CNF-GNP/SPE) were analyzed in a recent study. This type of sensor was studied in order to evaluate electrochemical behaviour, but also as a possible support for tyrosinase immobilization. Electrochemical characterization and qualitative and quantitative detection of ferulic acid in cosmetics represented the main purposes of developing this biosensor.

In order to prepare the biosensor, the tyrosinase enzyme was immobilized on the surface of the CNF-GNP/SPE electrode by using the casting technique, followed by crosslinking with glutaraldehyde. A solution of tyrosinase enzyme in 0.01 M PBS (pH 7.0) with a concentration of 50 ug × µL^−1^ was prepared for this procedure, then 10 µL of enzyme solution was added to the working electrode surface. For crosslinking, the CNF-GNP-Ty/SPE biosensor was placed on the top of a container with a 2% glutaraldehyde solution for 1 min. The biosensor was left for drying at room temperature and was next stored at 4 °C until use. This new biosensor allowed low values for the limit of detection (2.89 × 10^−9^ mol·L^−1^) and the limit of quantification (9.64 × 10^−9^ mol·L^−1^) respectively, which showed that the electroanalytical method is feasible for quantifying ferulic acid in real samples. The results obtained were validated by means of the infrared spectrometric method [53].

In another study, a variety of Yerba mate samples were analyzed to determine the polyphenol content, expressed as chlorogenic acid equivalents. The extracted chlorogenic acid, expressed as mg × g^−1^, caffeic acid and other phenolic compounds were evaluated by amperometry (using the biosensor), the Folin–Ciocalteu method and high performance liquid chromatography (HPLC). The results were similar.

The biosensor was obtained by immobilizing tyrosinase on the surface of a vitreous carbon electrode prepared in advance by polishing, rinsing and drying. The enzyme was dissolved in deionized water in order to obtain a concentration of 10 μg·μL^−1^. The solution was divided into volumes of 2.5 μL. Then, a 1/10 dilution with 25% glutaraldehyde solution in 50 mM phosphate buffer, pH 7.00 was made. Tyrosinase immobilization was performed by adding 1.5 μL of tyrosinase solution and 1.5 μL of 2.5% glutaraldehyde solution to the electrode surface. The mixture was allowed to dry for 1.5 h at room temperature (20 ± 2 °C) for polymerization. The enzymatic electrodes were rinsed with deionized water and stored in 50 mM phosphate buffer, pH 7.00 at room temperature for at least 2 h before use.

Optimal results were obtained for the detection of polyphenols content from Yerba mate with this biosensor, the procedure being easy, cheap and implying a minimum level of pollution [122].

An important improvement of electrochemical properties may be obtained by depositing a layer of conductive polymers (CP) on the electrode surface, thus favoring the immobilization of the enzyme [123,124]. CP electrodeposition and/or enzyme immobilization may be achieved by potentiostatic and/or galvanostatic methods or by superimposing sinusoidal voltages (SV) [125,126].

The SV method requires a shorter working time, being feasible for the construction of miniaturized biosensors for different applications. Moreover, the thickness of the electrodeposited biocomposite layer may be controlled when using SV, and the developed biosensors have high stability, repeatability and precision with a low detection limit [127].

In 2018, Garcia-Guzman and his team developed a poly biosensor (3,4-ethylenedioxythiophene) -tyrosinase/Sonogel-carbon (PEDOT-Tyr/SNGC) in order to analyze beer and wine products. This biosensor involved a new method of sinusoidal electrodeposition (SC) for enzyme immobilization. The biosensors were characterized electrochemically, by using cyclic voltammetry and electrochemical impedance spectroscopy.

The development of PEDOT-Tyr/SNGC biosensors was performed by using an aqueous solution which contained, in an optimized proportion, 3,4-ethylenedioxythiophene (EDOT), Tyr and phosphate buffer solution (PBS) with pH 7. The electrochemical procedure was performed in the next stage whereby sinusoidal currents (SC) with a single sine wave were superimposed over a direct current. An electrodeposition time of 300 s was used in order to cover the electrode surface with PEDOT-Tyr. The biosensors were washed with ultrapure water and used in electroanalytical applications. The polyphenol index was evaluated for several types of beers (alcoholic and nonalcoholic) and wines. Caffeic acid was used as the reference polyphenol. The obtained parameters (sensitivity, detection limit, and correlation coefficients) had good values, according to the studies in the field, proving the good electrochemical properties of the biosensor [128].

In another study, polyaniline (PANI) acted as an immobilization matrix of enzymes and conductive material, which had the role of amplifying the electrochemical signal.

The device was developed by incorporating Tyr into SWCNTs and PANIs on the surface of a vitreous carbon electrode (GCE). The analytical performance was evaluated by detecting the catechol, as well as an important hydroxycinnamic acid, i.e., caffeic acid, by using CV.

After a careful preparation of the SWCNT-modified GCE electrode, the EDC coupling agent (1-ethyl-3-[3-(dimethylamine) propyl] carbodiimide) was added dropwise together with Tyr, immobilizing the enzyme on the surface of SWCNTs/GCE. The last step implied PANI dispersal in a 0.1 M PBS solution (pH 6.5) containing 0.5% glutaraldehyde, and ultrasonication for 1 h. The solution was added on Tyr-SWCNTs/GCE, finally obtaining a PANI/Tyr-SWCNTs/GCE sandwich biosensor. The biosensor had good sensitivity, repeatability and stability and good ability to quantify caffeic acid in the tested samples [129].

Nano-fibrous membranes are nanostructures with specific surface area and high porosity. These characteristics are important for enzymatic immobilizations and efficient transport of the analytes to the electrode surface [130]. By means of this nanostructure, a tyrosinase-modified electrode was developed, which was used as an amperometric biosensor for the detection of phenolic compounds in food. Among the phenolic compounds analyzed, a hydroxycinnamic acid, i.e., caffeic acid, was also found. The enzyme tyrosinase was immobilized by dripping using, as a support, a vitreous carbon electrode covered by a membrane, polyamide nanofiber, prepared by electrospinning. This nanostructured coating influenced the permeability of phenols depending on the pH of the solutions and their dissociation constants. The performance and characteristics of the biosensor were evaluated by chronoamperometry in standard solutions and in real samples.

**Figure 6:**
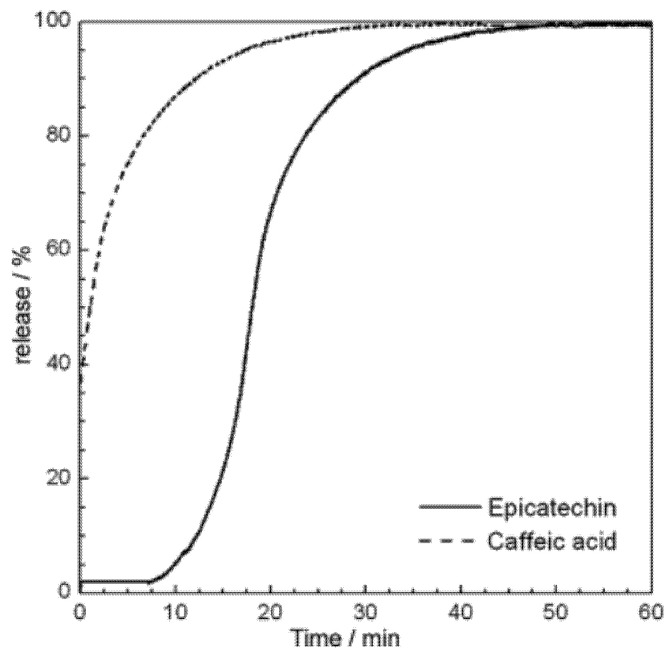
Epicatechin and caffeic acid release profiles of dry microcapsules obtained by spraying in an aqueous solution Reprinted from [130] with permission of publisher.

Figure 6 shows the epicatechin and caffeic acid release profiles in aqueous solutions from polymeric microcapsules. The total amount of epicatechin and caffeic acid was released after 40 and 30 min of stirring. These results were confirmed by colorimetric analysis (Folin–Ciocalteu method).

The biosensor may be successfully used, among others, for real-time release kinetics monitoring of phenols encapsulated in polymeric microcapsules [130].

Extra virgin olive oil is one of the foods with a high HC content. These compounds are largely responsible for the aroma, taste, and protection of the oil against oxidation. Given the importance and widespread consumption of this food, researcher S. Nadifiyine and his team set out to determine the phenolic compounds, including caffeic acid, from samples of virgin olive oil. Two tyrosinase-based biosensors were compared for this analysis, one supported by a graphite paste electrode (CPE-Tyr) and the other by a carbon black paste electrode (CBPE-Tyr). The content of phenolic compounds in olive oil was analyzed by reference to the activity of a CA solution before and after the injection of 250 mL of olive oil extract.

Enzyme immobilization was performed by crosslinking. A volume of 15 μL of Tyr solution (170 IU/mL) was mixed with 7.5 μL of bovine serum albumin (BSA) (1%) and 7.5 μL of 0.25% glutaraldehyde. In a short time interval (approximately 10 s), a volume of 7.5 μL of this mixture was sprayed on the surface of the electrodes, obtaining the CPE-Tyr and CBPE-Tyr biosensors. CBPE-Tyr reacted with a wide spectrum of phenolic compounds and showed a good correlation coefficient with the Folin–Ciocalteu colorimetric method for phenol determination in olive oil [131].

The biosensors based on tyrosinase are efficient for the sensitive detection of HCs in different type of samples and the results were validated by means of sprectrophotometric or chromatographic methods. The development of portable devices based on this type of biosensors could have an important impact in control quality and screening analysis.

### 5.2. Laccase-Based Enzymatic Biosensors

Discovered in 1883 in the *Rhus vernicifera* tree, laccase is one of the first enzymes described in domain-specific studies. Ten years later, G. Bertrand isolated this R. succedanea enzyme from other members of the Anacardiaceae family (e.g., *Mangifera indica, Schinus molle, Pistacia palaestina, Pleiogynium timoriense)* [132]. The fact is now known that this enzyme, which contains copper ions, has a number of unique catalytic properties. Laccase belongs to the family of multi-copper oxidases (MCO), a group of enzymes which includes numerous enzymes with different specificities for different substrates and various biological functions. Laccase consists of a group of four copper atoms (type 1 copper; type 2 copper and two type 3 copper atoms) which form the active site of the enzyme [133].

**Figure 7:**
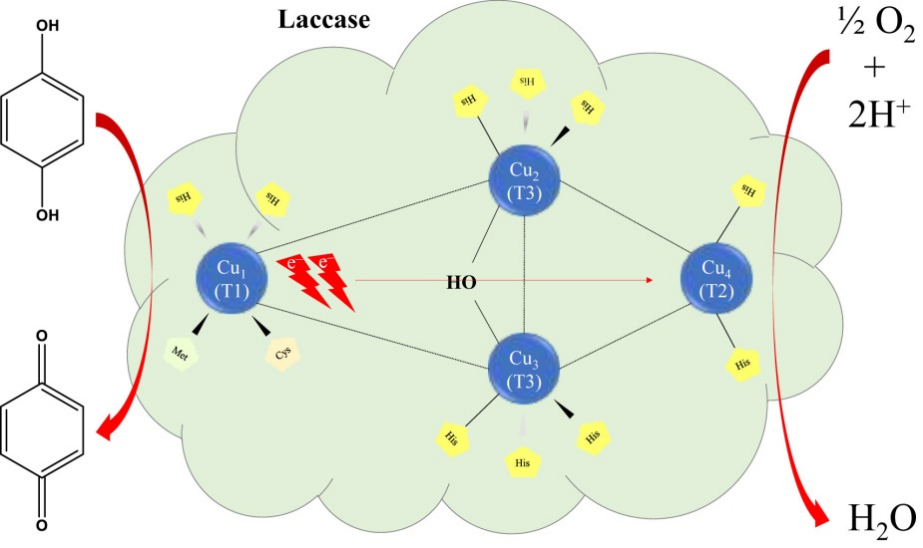
Schematic representation mechanism of substrate oxidation by Lac. Reprinted from [34,137] with permission of publisher.

Laccase interacts with ortho and para-diphenol groups, including mono-, di-, and polyphenols, aminophenols, methoxyphenols, aromatic amines and ascorbate, with the simultaneous reduction of four-electron oxygen in water [134]. The catalytic mechanism of laccase begins with the donation of an electron to the substrate by the T1 copper site, followed by an internal electron transfer from the reduced T1 to T2 and T3 copper sites. T3 copper functions as a two-electron acceptor in the process of aerobic oxidation, in which the presence of T2 copper is required. The reduction of oxygen in water takes place in groups T2 and T3 and implies a peroxide intermediate [135]. This oxidation mechanism of the substrate by lacasse is schematically represented in Figure 7.

Laccases are unable to directly oxidize non-phenolic substrates or large molecules with high redox potential [134]. Under the circumstances, mediators are required which form, together with laccase, intermediates able to produce a high redox potential able to indirectly oxidize non-phenolic substrates, increasing the range of compounds oxidizable by laccase [136,137].

Laccase has been used in biosensors based on metal nanoparticles, carbon nanomaterials, polymers and biopolymers (chitosan) or various membranes such as Nafion. The synergistic combination of nanomaterials and laccase which increases the performance of electrochemical biosensors [138] is highly relevant. An appropriate matrix of nanomaterials/metal nanoparticles and bovine serum albumin/β-cyclodextrin/bacterial cellulose may be made in order to improve biosensors stability [139,140,141].

These matrices provide a favourable environment, similar to the physiological one, which leads to high stability and strength. These sensors have been used, successfully, for routine determinations in pharmaceutical samples [139], as well as for hydroquinone determination in water samples [34,140].

A similar technique was used by S. Litescu and his team in their study devoted to secondary metabolites of polyphenols. A laccase-based biosensor was developed by specific adsorption on various working electrodes and stabilization with a membrane containing 0.1% Nafion.

The screen-printed working electrodes (Au-SPE, C-SPE) were modified by pouring a volume of 3 to 5 mL of laccase from a stock solution. The sensors were dried quickly and the Nafion membrane was added to immobilize the enzyme. After a series of optimization studies, an optimal consistency of the membrane was obtained which allowed the crossing of polyphenols in 0.1%. The biosensors were maintained at 4 °C for 12 h before their first use and stored under the same temperature conditions on a layer of silica gel to avoid damage by wetting.

The electrode was characterized in terms of response time, sensitivity, linearity range, detection limit, pH dependence, interference, and long-term stability. Hydroxycinnamic acids such as rosmarinic, caffeic, and chlorogenic acids were among the substances identified and tested. The optimized biosensor proved very good performance and was used successfully in real samples [141].

The integration of gold nanoparticles (AuNPs) into multi-walled carbon nanotubes (MWCNT) and their addition on graphene-based screen-printed electrodes (GPH) was the method approached by Favero et al. so as to improve the electrochemical properties of nanomaterials and efficient laccase immobilization. In order to prepare the biosensor, the GPH and MWCNT screen-printed electrodes were modified by depositing, on the electrode surface, 2 μL colloidal solution of AuNPs 1.4 × 10^−3^ μM, 4 μL of PVA-SbQ photopolymer, and 3 μL of a solution containing 0.306 U/mL of tyrosinase. Following deposition, the electrodes were left under a UV–Vis lamp for 20 min to immobilize the electrode surface.

**Figure 8:**
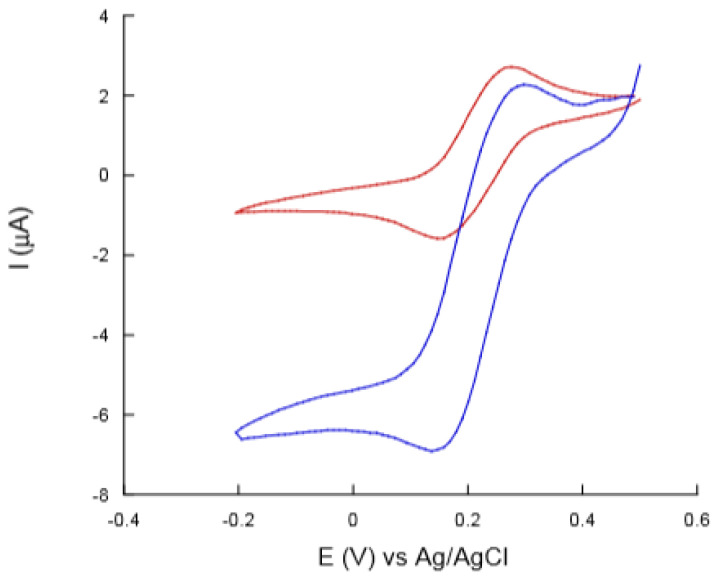
Cyclic voltammograms for 0.5 mM caffeic acid solution onto a MWCNTs-poly(vinylalcohol) (PVA)-AuNPs before (red curve) and after (blue curve) laccase immobilization [92].

The characterization of the modified electrode surface was performed by cyclic voltammetry. The results showed that the use of AuNPs to modify both graphene and MWCNT screen-printed electrode surfaces increases the electrochemical performance of the electrodes. Both electrodes proved effective in immobilizing Tyr. The electrochemical properties and detection capacity of the biosensor were investigated by using phenolic compounds, including caffeic acid. Figure 8 shows the cyclic voltammograms obtained with this sensor, before and after enzyme immobilization, tested by immersion in a 0.5 mM caffeic acid solution [92].

A topical study shows a precision modification of a carbon electrode, performed in a single step in which laccase is immobilized during the potentiostatic deposition of a thin film of polydopamine (ePDA). The morphology, wettability, optical, and electrochemical properties of the modified electrodes were studied by atomic force microscopy, goniometry with water contact angle, ellipsometry, and cyclic voltammetry.

The results point to the fact that laccase is immobilized and evenly distributed in the ePDA matrix and the redox behaviour of the polymer is not significantly affected. The procedure is fast and efficient and has been implemented on disposable graphite electrodes, with the aim of detecting phenolic compounds: caffeic, rosmarinic, and gallic acids. The catalytic performance of the modified electrodes was evaluated in terms of reproducibility, sensitivity, limit of detection (LOD) and linearity range by using chronoamperometry. The results obtained were optimal, allowing content estimation of low molecular weight phenols from chestnut peel extracts and industrial waste [37].

In 2020, a biosensor was built based on graphite oxide, platinum nanoparticles (PtNPs) and biomaterials (botyrosphere = BOT) obtained from *Botryosphaeria rhodina* by achieving an optimal 1:6:1 mixture of PtNPs, BOT and laccase which was used to modify the electrode.

The previously prepared graphite oxide paste was placed in a Teflon^®^ tube. The graphite oxide paste electrode was next positioned vertically with the active surface up to allow the addition of the other biosensor component (PtNPs, laccase and BOT) by dripping onto the electrode surface. The biosensor was prepared as follows: 3 μL of the PtNPs suspension was added on the surface of the graphite oxide paste electrode which was dried at room temperature for 1 h. Then, 12 μL of BOT solution followed by 3 μL of laccase were added sequentially on the surface containing layered PtNPs in the next stage and the biosensor was allowed to dry at 4 °C.

The biosensor was applied to the voltammetric determination of chlorogenic acid (CGA) measured as 5-O-caffeoyl-quinic acid (isochlorogenic acid, 5-CQA). The biosensor response was linear (R^2^ = 0.998) for 5-CQA in the concentration range 0.56–7.3 µmol·L^−1^, with the detection and quantification limits of 0.18 and 0.59 µmol·L^−1^, respectively. The new biosensitive device was applied in subsequent quality control studies based on the determination of CGA content in special and traditional coffee samples. The results indicated that the special coffee samples had a significantly higher CGA content than the traditional ones. The analysis of the main components of the voltammetric signals of the prepared coffee samples showed that the laccase-based biosensor may be used to identify them with adequate accuracy [16].

Along the years, nanocomposite materials have been shown great interest due to their structure which imprints excellent optical, electrical and catalytic properties, as well as good synergistic effects between nanoparticles and conductive polymers. One of the main applications of nanocomposites is visible in the field of electrochemistry, their use for the development of biosensors improving analytical performance [142]

A nanocomposite system was prepared by R. Penu and his team and applied in the construction of an amperometric biosensor designed to determine the total polyphenolic content of propolis extracts. The nanocomposite system (Lacc-TESBA-ITO-NPs) was based on the covalent immobilization of laccase on functionalized tin oxide nanoparticles followed by morphological and structural characterization. The biosensor was obtained by dripping 300 mIU Lacc-TESBA-ITO-NPs on the surface of a screen-printed electrode (C-SPE) which was allowed to dry at 4 °C. A 0.1% Nafion membrane was used to stabilize the bio-component.

The analytical performance characteristics of the prepared biosensor were determined for rosmarinic acid, caffeic acid and catechol. The linearity range was 1.06 × 10^−6^–1.50 × 10^−5^ mol·L^−1^ for rosmarinic acid and 1.90 × 10^−7^–2.80 × 10^−6^ mol·L^−1^ for caffeic acid. The sensitivity and limit of detection obtained for caffeic acid were 141.15 nA·µmol^−1^·L^−1^ and 7.08 × 10^−8^ mol·L^−1^, respectively. The results obtained for the polyphenolic content of propolis extracts and those obtained by liquid chromatography with diode array detector proved highly similar [143].

**Figure 9:**
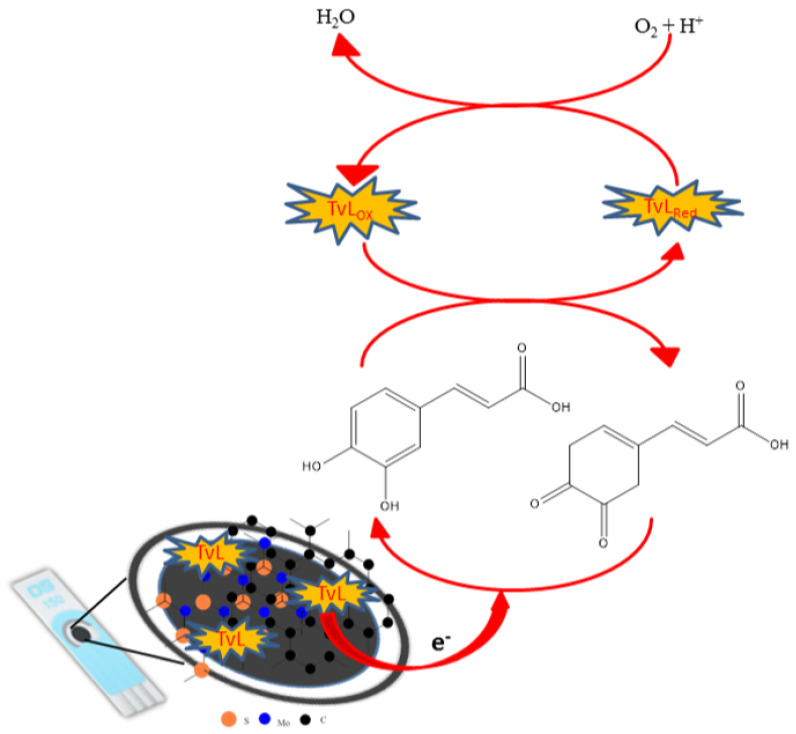
Construction and operating principle of the CSPE-MoS_2_-GQDs-TvL biosensor. Reprinted from [144] with permission of publisher.

Another type of nanocomposite consisting of molybdenum disulfide (MoS_2_) and quantum graphene particles (GQDs) was proposed as a support for laccase immobilization thanks to its giving excellent electroanalytical properties to the developed biosensor. Screen-printed carbon electrodes were used as a substrate for the biosensor due to their good conductivity, manufacturing reproducibility and low cost. First, a volume of 8 µL of suspension prepared from MoS_2_ (18 mg·L^−1^) was deposited on CSPE. After drying, 6 µL of suspension from GQDs was added dropwise. Finally, enzyme immobilization was performed by pouring 5 μL of a 58.23 U·mL^−1^ laccase solution onto the modified working electrode which was allowed to dry in a dehumidified chamber at 24 °C and was stored at 4 °C until use. Figure 9 describes the construction and operating principle of the CSPE-MoS_2_-GQDs-TvL biosensor. Basically, laccase immobilization at the electrode surface took place through the electrostatic interaction between the negatively charged laccase and the positively charged GQDs. The developed biosensor was tested by using cyclic voltammetry for the detection of caffeic acid in a concentration range between 0.38 and 100 µM, the results being a detection limit of 0.32 µM and a sensitivity of 17.92 nA·μM^−1^. The proposed biosensitive device has been successfully applied to determine the total polyphenolic content of red wine samples [144].

The biosensors based on laccase have the advantages of versatility to detect different compounds with high sensitivity and selectivity, without major drawbacks. These devices could be implemented in routine analysis.

Table 1 presents the main enzymatic biosensors, analytes of interest, detection technique, enzyme immobilization technique and main electroanalytes.

The support electrodes most commonly used were screen-printed sensors based on quantum graphene particles, single- or multilayer carbon nanotubes, or electrodes made of vitreous carbon. As regards the changes to the contact surface, metal nanoparticles (AuNPs, and PtNPs) were the most common ones. Many authors have also used various conductive polymers (PEDOT, PANI, and polydopamine), nanomaterials (molybdenum disulfide, graphene quantum particles), biomaterials (botyosphere), or membranes capable of streamlining the enzyme adsorption (Nafion, and chitosan). Nanomaterials used as modifiers have led to an increase in the surface area available for enzyme immobilization, a better electrical conductivity and an increased electron transfer rate.

The techniques for laccase and tyrosinase immobilization most often used were the casting technique followed by crosslinking, adsorption and electrodeposition. Regarding electrochemical transduction, chronoamperometry was predominantly used, followed by cyclic voltammetry, differential pulse voltammetry, and square wave voltammetry. In most studies, the validation of the results obtained with the developed biosensors was performed by using the Folin–Ciocalteu spectrophotometric method or with HPLC. Representatives of hydroxycinnamic acids of great interest for analysis were: caffeic acid, chlorogenic acid, and rosmarinic acid. Ferulic acid was analyzed at a lower frequency.

Laccase or tyrosinase biosensors have a wide applicability in areas such as: health, food or environmental control. In a typical laccase-catalyzed reaction, a phenolic substrate is subjected to single-electron oxidation to give an aryloxy radical. This active species is converted to quinone in the second stage of oxidation [145].

Tyrosinase catalyzes two different oxygen-dependent reactions: o-hydroxylation of monophenol to produce o-diphenol (cresolase activity) and subsequent oxidation of o-diphenol to o-quinone (catecholase activity). Therefore, laccase or tyrosinase-based biosensors have high specificity for the detection of diphenolic and monophenolic compounds, respectively, and traditional methods of electrochemical transduction have been continuously improved with functionalized nanomaterials.

## 6. Conclusions

Laccase and tyrosinase electrochemical biosensors have provided excellent results for the detection of hydroxycinnamic acids. Analyzing domain specific studies, the fact may be noticed that various nanomaterials and nanocomposites have been used to streamline the process of enzyme immobilization as well as to improve analytical performance.

Studies on laccase- or tyrosinase-based biosensors for the electrochemical detection of three other HCs, i.e., synaptic, p-coumaric acid, and cinnamic acid, were not identified in the present review. Therefore, further research needs to be considered for the detection and quantification of other compounds in the class of hydroxycinnamic acids by using innovative enzyme biosensors based on nanomaterials, and the actual samples analyzed should belong to several applied fields of interest, such as food, pharmaceutical or cosmetics. The detection of HCs in extra virgin olive oil samples is particularly interesting because these compounds have beneficial effects on the human body and may be used, at the same time, as authenticity biomarkers.

The main challenges of enzymatic biosensors could be their applicability on the market or in real life. This involves the development of miniaturized, minimally invasive biosensors, produced by bioengineering. Researchers must also take into account the fact that real samples, especially biological fluids have a complex composition, in which different compounds can interact, reducing redox activity and thus the electrochemical signal. Laccase or tyrosinase biosensors are able to determine the total content of phenolic compounds but also a specific phenolic compound, depending on the structural affinity of the enzyme. The technique by which the enzyme is captured on the support is also very important, as well as the materials used. Limitations related to reduced enzyme activity or surface contamination could be reduced or avoided by the use of complex nanocomposites with anti-fouling ability capable of acting synergistically with the enzyme.

It is necessary that future studies focus on methods of developing biosensors that avoid contamination of the active surface and interference caused by chemicals present in the sample to be analyzed as well as the possibility of simultaneous detection of several analytes in different applications. The development of third generation biosensors, the miniaturization of devices and the possibility of using a minimum amount of samples and reagents, could be solutions for accurate and fast results obtained on site.

## Figures and Tables

**Table 1:** The main biosensors based on tyrosinase and laccase, respectively, for the detection of HCs, electrochemical technique, enzyme immobilization technique, linearity range, and detection limit.

Sensitive Material	Analyte	Detection Technique	Immobilization Technique	Linearity Range	LOD	Ref.
Tyrosinase						
Tyr-AuNPs-SPCEs	CA	Amp	Casting and crosslinking	2.5–12.5 μM	2.3 μM	[26]
CNF-GNP-Ty/SPE	FA	CV	Casting and crosslinking	0.1–1.6 μM	2.89 × 10^−9^ mol·L^−1^	[53]
Electrode of glassy carbon modified with tyrosinase	CGA	Amp	Casting and crosslinking	70–400 μM	2.52 μM	[122]
	CA			20–200 μM 50–370 μM	2.68 μM 1.82 μM	
PEDOT-Tyr/SNGC	CA	ChronoAmp	Electrodeposition with sinusoidal current	10–300 μM	4.33 μM	[128]
PANI/Tyr-SWCNTs/GCE	CA	CV	Casting, coupling agent EDC and crosslinking	10^−7^–4.7 × 10^−4^ M	6 × 10^−8^ M	[129]
Tyr-biosensor	CA	ChronoAmp	Droping + membrane from poliamidic nanofiber	-	-	[130]
CBPE-Tyr	CA	Amp	Atrappment and crosslinking	-	-	[131]
MWCNTs-PVA-AuNPs-Tvl	CA	ChronoAmp CV	Casting	1–100 μM	0.5 μM	[92]
Laccase						
ePDA-Lac	CA	ChronoAmp	Casting and simultaneous potentiostatic deposition of ePDA (matrix)	1–50 μM	0.14 μM	[37]
	RA			1–20 μM	0.09 μM	
PtNPs-BOT-Lac/GO	CGA (5CQA)	SWV ChronoAmp CV	Casting use of static mixture PtNPs:BOT:Lac	0.56–7.3 μmol × L^−1^	0.18 μmol·L^−1^	[16]
Lacc-TESBA-ITO-NPs	RA	CV	Covalent immobilization	1.06 × 10^−4^–1.5 × 10^−5^ mol × L^−1^	9 × 10^−7^ mol × L^−1^	[143]
	CA			1.9 × 10^−7^–2.8 × 10^−6^ mol × L^−1^	7.08 × 10^−8^ mol·L^−1^	
CSPE-MoS_2_-GQDs-Tvl	CA	CV	Casting Electrostatic interaction	0.38–100 μM	0.32 μM	[144]
Lacc–CS–MWCNT	CA	CV	Electrodeposition	7.35 × 10^−7^–1.05 × 10^−5^	1.51 × 10^−7^ M	[22]
	RA			9.1 × 10^−7^–1.21 × 10^−5^	2.33 × 10^−7^ M	
	CGA			7.93 × 10^−7^–6.71 × 10^−6^	1.61 × 10^−7^ M	
Laccase-Nafion biosensor Lc-DAD-ESI-MS	CA	ChronoAmp	Adsorption Stabilization with Nafion membrane	3 × 10^−6^–1.5 × 10^−5^ mol × L^−1^	2.5 × 10^−6^ mol·L^−1^	[141]
	CGA				2.8 × 10^−6^ mol·L^−1^	
	RA				2.4 × 10^−6^ mol·L^−1^	

CA—caffeic acid, FA—ferulic acid, RA—rosmarinic acid, CGA—chlorogenic acid, CQA—5-O-caffeoyl-quinic acid; Tyr-AuNPs-SPCEs—screen-printed electrode modified with Au nanoparticles and tyrosinase; CNF-GNP-Ty/SPE—screen-printed electrode based on carbon nanofibers modified with Au nanoparticles and tyrosinase; PEDOT-Tyr/SNGC—sonogel-carbon electrode modified with Poly (3,4-ethylenedioxythiophene) and tyrosinase; PANI/Tyr-SWCNTs/GCE—glassy carbon electrode modified with single walled carbon nanotubes, polyaniline and tyrosinase; CBPE-Tyr—tyrosinase-modified carbon black electrode; Lc-DAD-ESI-MS—biosensor based on laccase and Nafion membrane; MWCNTs-PVA-AuNPs-Tvl—screen-printed electrode based on graphene modified with multilayer carbon nanotubes, gold nanoparticles and laccase; ePDA-Lac—biosensor based on laccase and polydopamine film; PtNPs-BOT-Lac/GO—biosensor based on graphite oxide, platinum nanoparticles and a biomaterial obtained from *Botryosphaeria rhodian;* Lacc-TESBA-ITO-NPs –screen-printed electrode based on carbon functionalized with indium oxide nanoparticles and triethoxysilylbutyraldehyde (TESBA) and laccase; CSPE-MoS2-GQDs-Tvl—electrode modified with molybdenum disulphite, graphene nanoparticles and laccase (Trametes versicolor); Lacc–CS–MWCNT—biosensor based on multilayer carbon nanotubes, chitosan, and laccase.
